# LPS-Primed Release of HMGB-1 from Cortical Astrocytes is Modulated Through PI3K/AKT Pathway

**DOI:** 10.1007/s10571-015-0223-5

**Published:** 2015-06-27

**Authors:** Ze-Feng Xie, Gang Xin, Yan-Xuan Xu, Yun Su, Kang-Sheng Li

**Affiliations:** 1 The First Affiliated Hospital of Shantou University Medical College, Shantou, Guangdong China; 2 Department of Microbiology and Immunology, Shantou University Medical College, 22, Xinling Road, Shantou, Guangdong China

**Keywords:** Astrocyte, HMGB-1, LPS preconditioning, PI3K/AKT pathway

## Abstract

Studies have shown that LPS-preconditioned tolerant state could protect against brain injury to subsequent challenges. We hypothesized astrocytes were directly involved in the readjustment to confer neuroprotective effects with LPS pretreatment. High-mobility group box 1(HMGB-1) from LPS-preconditioned astrocytes, presumably serving as a positive regulator, might contribute to the favorable preconditioned effects. Furthermore, a potential cellular pathway (PI3K/AKT pathway), has been proposed for the active regulation of LPS-primed reactive astrocytes to secrete HMGB-1. In the present study, we used a low concentration of LPS to directly prime the astrocytes in vitro, and the subsequent astrocytic reactions, including cytokine secretion, the expression of transcription factors, and the release of HMGB-1 were examined after the blockade of the PI3K pathway. The data showed that LPS preconditioning could reduce some capacity of astrocytes to subsequent challenge in vitro. PI3K/AKT pathway was partially involved in the modulation of the release HMGB-1 from reactive astrocytes. These findings offer direct evidence supporting the flexible roles of astrocytes in mediating LPS-primed neuroprotection, and highlight additional targets for future attempts to modify the protective effects of astrocytes through LPS preconditioning.

## Introduction

Classically, preconditioning with a relatively low concentration of lipopolysaccharide (LPS) previously (Beeson 1947; Virca et al. 1989) attenuates the responses to LPS activation in animals and humans, and in cultured macrophage/monocytes (in vitro). The pre-exposure of low doses of harmful stimuli (such as LPS) elicit hyporesponsiveness for protection against subsequent injurious challenges (Virca et al. 1989). Recent studies have demonstrated that a tolerant state could also protect against brain injury to subsequent challenges including focal ischemia, cortical spreading depression, brief episodes of seizure, or exposure to MOG33-35 (Simon et al. 1993; Kobayashi et al. 1995; Towfighi et al. 1999; Buenafe and Bourdette 2007). Although the mechanisms involved in LPS preconditioning are incompletely understood, transient cellular hyporesponsiveness has been associated with the decreased inflammatory response, and the reprogramming of cellular signal pathways in response to LPS priming (Vartanian and Stenzel-Poore 2011). Within the brain, the inflammation due to immune or neural cells, including antigen presenting cells (APC) and the microglia, was thought to be readjusted to confer neuroprotective effects in response to LPS pretreatment (Rosenzweig et al. 2004).

Astrocytes, the most numerous “brain-resident” neural cell types in the mammalian brain, have diverse constitutions and functions, which may presumably play roles in the neuroprotective context of LPS-primed hyporesponsiveness. Generally, reactive astrocytes might contribute to neuronal remodeling and recovery (Silver and Miller 2004). Reactive astrocytes after stroke or brain injury might act as APC or subserve the inflammatory reaction via the release of various mediators, including nerve growth factor, interleukins, NO, and so on (Horner and Gage 2000; Chen and Swanson 2003). Similarly, a nonhistone DNA-binding molecule, called high-mobility group box-1 (HMGB-1), was also secreted from the reactive astrocytes (Passalacqua et al. 1998; Kim et al. 2006, 2008). HMGB-1, a pivotal protein might serve as a positive regulator in coordinating some cell processes, such as inflammation, proliferation, migration, and survival (Wang et al. 2004; Sun and Chao 2005; Yang et al. 2005; Ulloa and Messmer 2006). Studies have shown that HMGB-1 promotes proliferation and sprouting in endothelial cells (Treutiger et al. 2003; Mullins et al. 2004; Schlueter et al. 2005). This protein also facilitates neurite outgrowth, up-regulates synaptic proteins, and sustains cell survival in neurons (Huttunen et al. 1999, 2002; Srikrishna et al. 2002). Hence, during brain injury and inflammation, HMGB-1 might be secreted from reactive astrocytes as a potential mediator in the regulation of cerebral remodeling and recovery, involving the preconditioned favorable effects to subsequent injurious challenge.

To date, the responses of astrocytes to LPS pretreatment, and the mechanism that regulates the release of HMGB-1 from astrocytes in response to LPS preconditioning in vitro have not been completely characterized. In the present study, we initially investigated the mouse astrocytic reaction through LPS preconditioning and evaluated HMGB-1 expression from reactive cortical astrocytes. Additionally, during induction, given that several potential cellular pathways could mediate LPS-primed tolerance, we probed to PI3K/AKT pathway which was reported to regulate negatively expression of some inflammatory genes previously in a LPS challenge (Diaz-Guerra et al. 1999; Cantley 2002; Guha and Mackman 2002; Luyendyk et al. 2008), so as to further understand mechanisms of HMGB1 in response to low-dose LPS pre-exposure in astrocytes. These findings showed that the release of HMGB1 from LPS-primed astrocytes was regulated in part, through PI3K/AKT signaling.

## Methods

### Cell Culture

Using the modified technique described by McCarthy and de Vellis (McCarthy and de Vellis 1980), primary astrocyte culture was prepared from the cerebral cortices of neonatal C576L/BJ mice (less than 1-day-old) (Guangzhou, China). Briefly, dissociated cortical cells were suspended in Dulbecco’s modified Eagle’s medium/nutrient mixture F12 culture medium (DMEM/F12, 1:1) (Gibco, USA) supplemented with antibiotics (100 U/ml penicillin, 100 g/ml streptomycin, and 0.25 g/ml of amphotericin B; Gibco, USA), and 10% (v/v) heat-inactivated fetal bovine serum (FBS) (Beyotime, China), and were subsequently cultured in 75 cm^2^ flasks. The astrocytes were isolated and purified at 21 days after plating through trypsinization and shaking. Non-astrocytic cells including microglia and neurons were detached from the flasks after shaking and then removed through trypsinization. The purified astrocytes were reseeded onto uncoated 6-well plates or slide chambers at a density of 1 × 10^6^ cells/cm^2^. Thereafter, the following experiments were initiated after 24 h. In the culture, the cells were identified as astrocytes through astrocytic marker glial fibrillary acidic protein (GFAP) staining, with a flattened, polygonal morphology. All procedures were performed in compliance with the Experimental Animal Management Bill of November 14, 1988 (Decree No. 2 of the National Science and Technology Commission, People’s Republic of China) and according to the National Institutes of Health Guide for the Care and Use of Laboratory Animals (NIH Publications No. 80-23), revised in 1996.

### LPS Treatment

After the purification of astrocytes for 24 h, the cells were preconditioned with 0.01 μg/ml LPS in DMEM/F12 medium without FBS and incubated at 37 °C for 18 h. Thereafter, the cells were re-treated with 1 μg/ml LPS for 24 h, and subsequently the astroglial cells and supernatants were harvested. In this experiment, non-treated group, single dose of LPS-treated groups (0.01 and 1 μg/ml) were included. The preconditioning paradigm described above using LPS above was modified according to previous tolerance literature in which the preconditioning stimulus was a low dose of LPS (Cross 2002; Leung et al. 2009; Marsh et al. 2009; Leung et al. 2012). Moreover, to determine the role of the PI3K/AKT pathway on the expression of HMGB-1, one group of astrocytes was treated with the PI3K/AKT inhibitor of LY294002 (20 μM) for 2 h before low dose of LPS preconditioning as previously described.

### Immunofluorescence

After washing with ice-cold PBS (pH 7.4), the cells were fixed using 4 % paraformaldehyde for 10 min. Subsequently, the cells were further washed three times in PBS containing 0.1 % Triton X-100, followed by an incubation with 3 % bovine serum albumin (BSA) in PBS for 30 min. Thereafter, the cells were incubated with primary antibodies against the astrocytic marker glial fibrillary acidic protein (GFAP; 1:100; Millipore) or HMGB-1 (1:200; Abcam) at 4 °C overnight. NF-κb activation was detected using the Nuclear Translocation Assay Kit, and the translocation of p65 NF-κb from cytoplasm to the nuclear revealed the activation of NF-κb. The cells were incubated with a primary antibody against p65 NF-κb (1:100; Cell Signaling Technology) at 4 °C overnight to determine the intracellular location of the protein. After washing with PBS, cells were incubated with goat anti-mouse IgG against GFAP with AlexaFluor488 (1:400; Beyotime) or goat anti-rabbit IgG against HMGB-1 conjugated with FITC (1:200; Beyotime) or goat anti-rabbit IgG against NF-κb conjugated with Cy3 (1:200; Beyotime) for 1 h at room temperature. Finally, the nuclei was counterstained with 4,6-diamidino-2-phenylindole (DAPI), and the coverslips were placed. Immunostaining was analyzed using a fluorescence microscope (Nikon) interfaced with a digital charge-coupled device camera and an image analysis system.

### Preparation of Nuclear and Cytoplasmic Extracts

After LPS treatment, astroglial cells were harvested and washed twice with cold PBS, and nuclear and cytoplasmic extracts were prepared using a protein extraction kit. We prepared a single cell suspension containing 1 × 10^6^ cells in 6-well plates and resuspended the cell pellet using 100 µl of protein extraction reagent A, followed by vortexing for 5 s at high speed and incubating on ice for 10 min. Subsequently, 5 µl of protein extraction reagent B was added, followed by vortexing again for 5 s at high speed and incubation on ice for 1 min. The cytoplasmic fraction was extracted through centrifugation at 16,000×*g* for 5 min at 4 °C. Thereafter, the pellet was resuspended in 25 µl of nuclear extraction reagent containing 1 µM phenylmethanesulfonyl fluoride (PMSF), followed by vortexing for 15 s at high speed, incubating on ice for 1 min, and vortexing again for 15 s at high speed. The same steps were repeated for 30 min, and the nuclear extract was prepared from the supernatant after centrifugation at 16,000×*g* for 5 min at 4 °C.

### Western Blot Analysis

Protein extraction solution (Beyotime) was added to the cultured cells. The protein concentration was determined using the Bradford assay (Bio-Rad). The cell lysates (20 µg per lane) were separated by SDS-PAGE and transferred to nitrocellulose membranes (Novex). The membranes were blocked in Tris-buffered saline containing 0.1 % Tween 20 and 5 % BSA for 90 min at room temperature, followed by incubation overnight at 4 °C with monoclonal anti-HMGB-1 (1:1000, Abcam), anti-NF-κb (1:1000, Cell Signaling Technology), monoclonal anti-*p*-AKT antibody (1:1000, Cell Signaling Technology), anti-AKT antibody (1:1000, Cell Signaling Technology), monoclonal anti-Histone H3 antibody(1:3000, Abcam), or monoclonal β-actin antibody(1:1000, Beyotime). After incubation with peroxidase-conjugated secondary antibodies (Beyotime) and visualization through enhanced chemiluminescence (Cell Signaling Technology), band intensity was determined using Image J software.

### Cytokine Assay

The levels of TNF-α or IL-6 released from mice astrocytes into the supernatants were determined using commercially available ELISA kits (Dakewei, China) according to the manufacturer’s instructions.

### Statistical Analysis

The data are expressed as the mean ± SEM. One-way ANOVA was used for the statistical analysis using SPSS 17.0 software. A *P* value < 0.05 was considered statistically significant.

## Results

### Astrocyte Culture and Identification

**Fig. 1:**
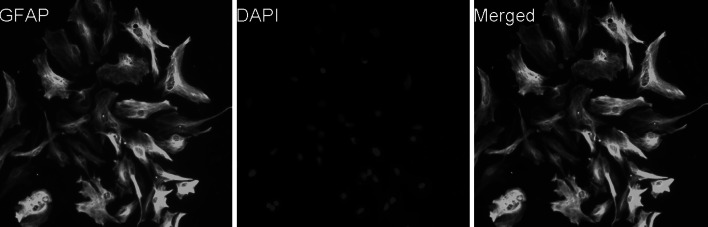
Astrocytes identification (×200). Immunofluorescence staining demonstrated the purity of astrocytes in our purified culture system. The cells in our purified system were stained for the astrocytic marker glial fibrillary acidic protein (GFAP; *green*). 97% of cells were identified as astrocytes; DAPI was used for staining for the nuclei (*blue*) (Color figure online)

Using a purified astroglial culture system, 97 % of cells stained for the astrocytic marker GFAP were identified as astrocytes (Fig. 1).

### Influence of the PI3K/AKT Pathway on TNF-α and IL-6 Expression from Cultured Astrocytes After LPS-Preconditioning

**Fig. 2:**
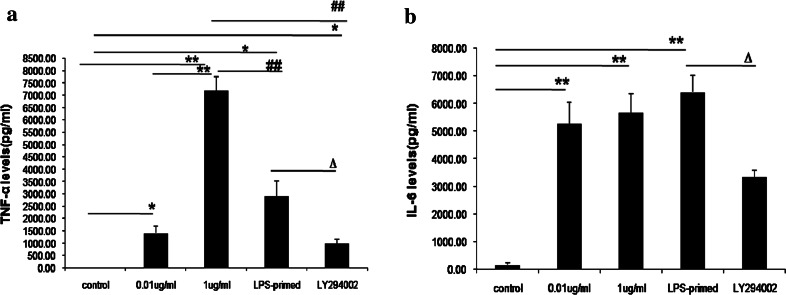
Influence of PI3K/AKT pathway on TNF-αand IL-6 levels in cultured astrocytes after LPS-preconditioning (*n* = 5). **a** Influence of PI3K/AKT pathway on TNF-α levels in cultured astrocytes with LPS priming(compared with non-treated group, * *P* < 0.05; ** *P* < 0.01; compared with the 1 μg/ml of LPS-treated group, ^*##*^ *P* < 0.01; compared with the LPS-primed group, ^*∆*^ *P* < 0.05); **b**. Influence of PI3K/AKT pathway on IL-6 levels in astrocytes in cultured astrocytes after LPS priming (compared with the non-treated group, ** *P* < 0.01; compared with the LPS-primed group, ^*∆*^ *P* < 0.05)

As shown in Fig. 2a and b, the levels of TNF-αand IL-6 in the supernatants were enhanced after 0.01 or 1 μg/ml of LPS challenge(*P* < 0.05, *P* < 0.01), compared with the control group. A smaller dose of 0.01 μg/ml of LPS pretreatment for 18 h could also increase TNF-α and IL-6 expression (*P* < 0.05, *P* < 0.01). However, the levels of TNF-α from LPS-pretreated astrocytes were lower than from those in response to LPS challenge at a dose of 1 μg/ml (*P* < 0.01). These findings indicated that a lower dose of LPS pretreatment can decrease some inflammatory cytokine secretion from cultured astrocytes with subsequent LPS exposure. Additionally, in our study, we used LY294002 to inhibit PI3K pathway, the data showed TNF-α and IL-6 secretion after LPS preconditioning were reduced significantly (*P* < 0.05, *P* < 0.01). The results suggested that the inhibition of PI3K/AKT pathway might exert suppressive effects on the release of inflammatory cytokines from cultured astrocytes, which might facilitate the roles of LPS priming.

### The Expression of NF-κb After LPS-Preconditioning and Influence of PI3K/AKT Pathway on LPS-Primed NF-κb Expression

**Fig. 3:**
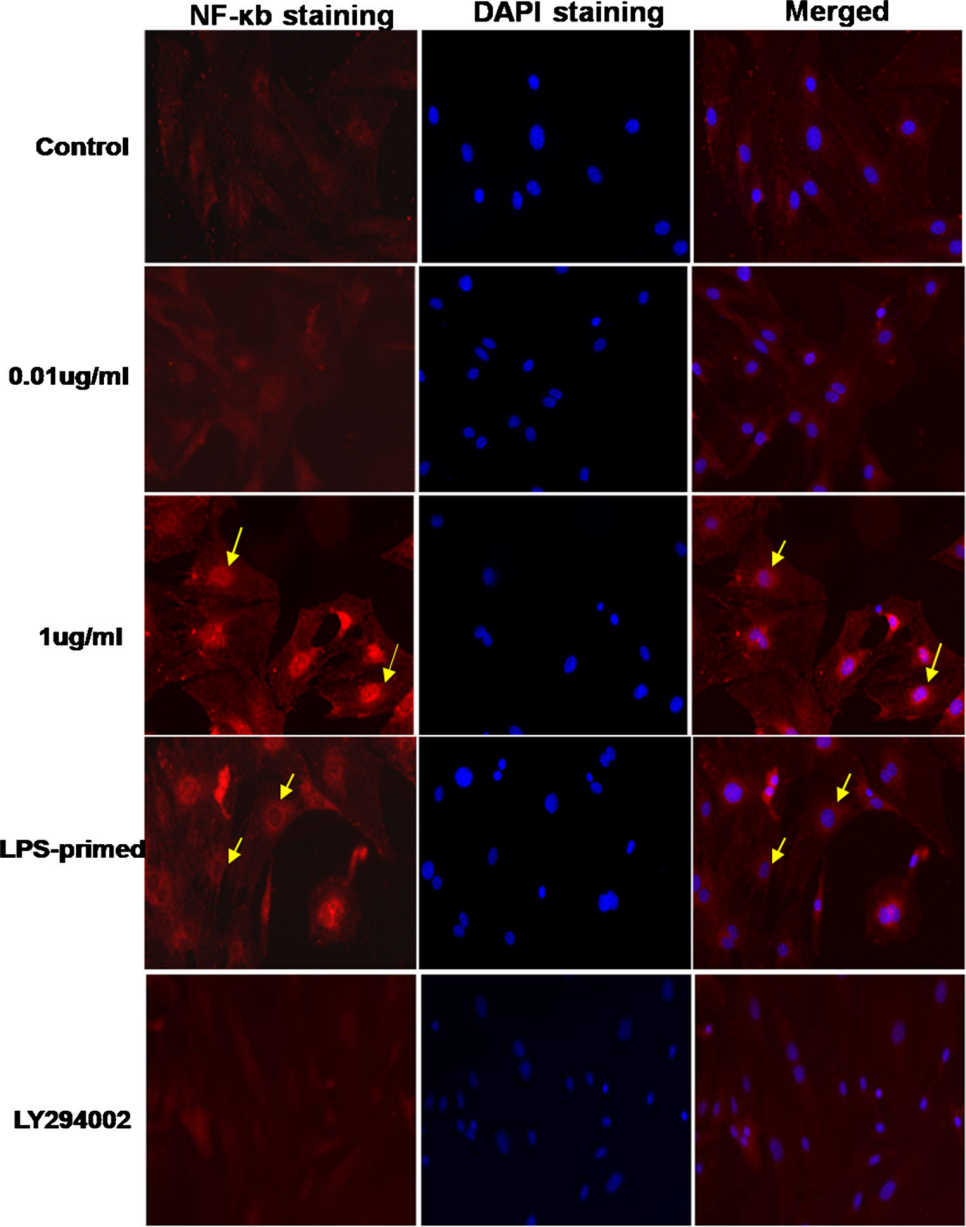
The expression of astrocytic NF-κb after LPS-preconditioning and influence of PI3K/AKT pathway on LPS-primed NF-κb expression. The astrocytes were preconditioned using 0.01 μg/ml of LPS for 18 h, followed by treatment with 1 μg/ml of LPS for 24 h. Fluorescence microscopy was used to observe the expression of astrocytic NF-κb (p65 NF-κb Ab-targeted NF-κB (*red*), and DAPI-targeted nuclei (*blue*)). The *arrowheads* indicated nuclear NF-κb. The Images are representative of three independent experiments (Color figure online)

**Fig. 4:**
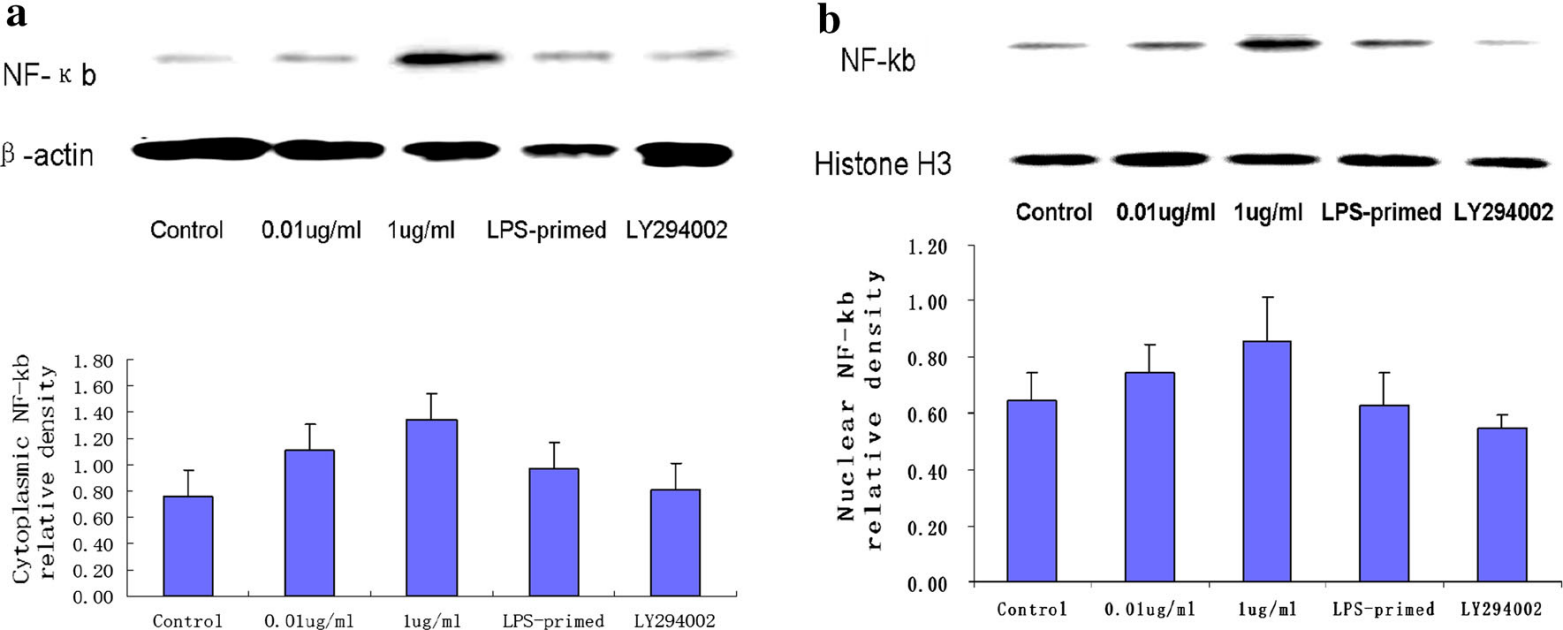
The expression of NF-κb in the cytoplasm and nuclei of LPS-primed astrocytes with LY294002 treatment. The astrocytes were preconditioned with a lower dose of LPS (0.01 μg/ml) for 18 h, followed by treatment with 1 μg/ml of LPS for 24 h. The cells were harvested for preparation of nuclear and cytoplasmic extracts to assess the nuclear and cytoplasmic NF-κb protein. * Western blot* analysis showed LPS preconditioning tended to reduce the nuclear expression of NF-κb with subsequent LPS exposure. In the cytoplasm, LPS-primed astrocytes showed no obvious changes in comparison with 1 μg/ml of LPS-treated group. Using LY294002 to inhibit PI3K/AKT pathway, NF-κb expression exhibited a reduction trend in both cytoplasm and nuclear. The * images* are representative of three independent experiments

The nuclear translocation assay kit was used for detection the activation of NF-κb. The translocation of p65 NF-κb from cytoplasm to nuclear reveals the activation of NF-κb. In non-treated group and 0.01 μg/ml of LPS-treated group, NF-κb localized predominantly in the cytoplasm, and 1 μg/ml of LPS-induced increase in astroglial expression of NF-κb was apparent in both the nuclei and cytoplasm. Nonetheless, with a lower dose of LPS priming, the expression of NF-κb in the nuclei was decreased (Fig. 3). These results suggested that LPS preconditioning could restrict LPS-induced NF-κb nucleocytoplasmic shutting toward the nuclei. We also used LY294002 for inhibition of PI3K/AKT pathway, the positive staining of NF-κb in astrocyte decreased in both cytoplasm and nuclei. Furthermore, we assessed astrocytic expression of NF-κb in the cytoplasm and the nuclei with LPS challenge via Western blot analysis. As shown in Fig. 4b, the results of Western blot showed LPS preconditioning tended to reduce the nuclear expression of NF-κb with subsequent LPS exposure, though there was no significant difference statistically. As for the expression of NF-κb in the cytoplasm, LPS-primed astrocytes showed no obvious changes in comparison with 1 μg/ml of LPS-treated group. Using LY294002, the inhibition of PI3K/AKT pathway tended to restrict the NF-κb expression in both cytoplasm and nuclei of LPS-primed astrocytes(Fig. 4a and b).

### Influence of the PI3K/AKT Pathway on HMGB-1 Expression in Astrocytes with LPS-Preconditioning

**Fig. 5:**
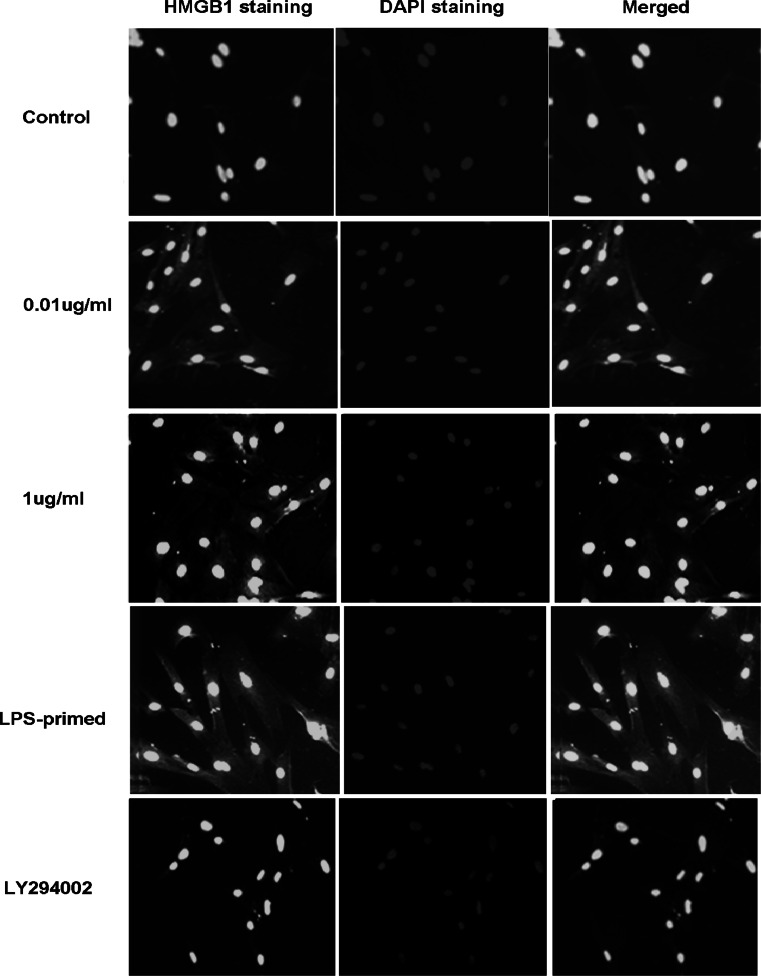
Influence of the PI3K/AKT pathway on the expression of HMGB-1 in astrocytes with LPS preconditioning. LPS-preconditioning increased the translocation HMGB-1 into the cytoplasm. With the treatment of LY294002, HMGB-1 translocation was inhibited. The cells were stained for the anti-HMGB-1 antibody (*green*), and for a nuclei stain DAPI (*blue*). Images are representative of three independent experiments (Color figure online)

**Fig. 6:**
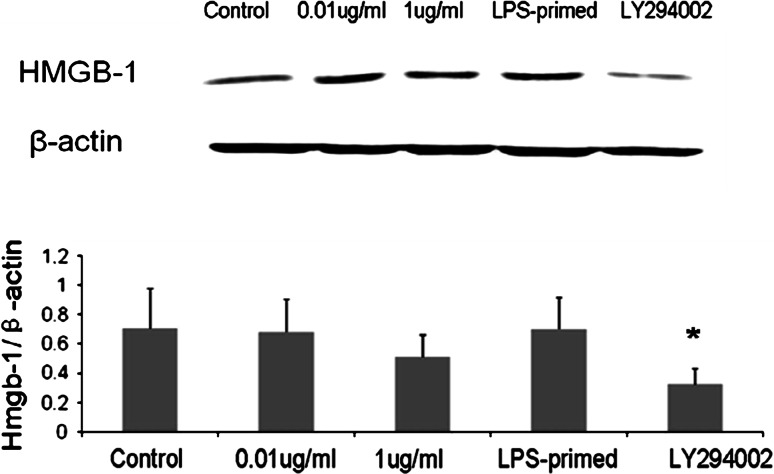
HMGB-1 expression of the LPS-primed astrocytes after the inhibition of the PI3K/AKT pathway. LPS (0.01 and 1 μg/ml) treatment tended to upregulate total HMGB-1 expression in astrocytes. LY294002 effectively reduced the expression of HMGB-1 in astrocytes (compared with LPS-primed group, * *P* < 0.05). The images are representative of three independent experiments

**Fig. 7:**
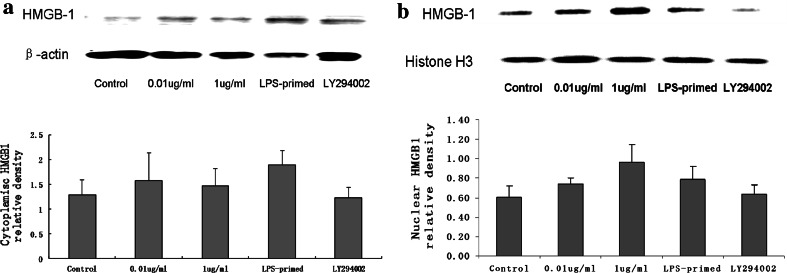
HMGB-1 expression in the cytoplasm and nuclei of LPS-primed astrocytes with LY294002 treatment. Western blot analysis revealed LPS preconditioning tended to enhance cytoplasmic HMGB-1. Using LY294002 to inhibit PI3K/AKT pathway, HMGB-1 expression of LPS-primed astrocytes showed a reduction trend in both cytoplasm and nuclei. The * images* are representative of three independent experiments

**Fig. 8:**
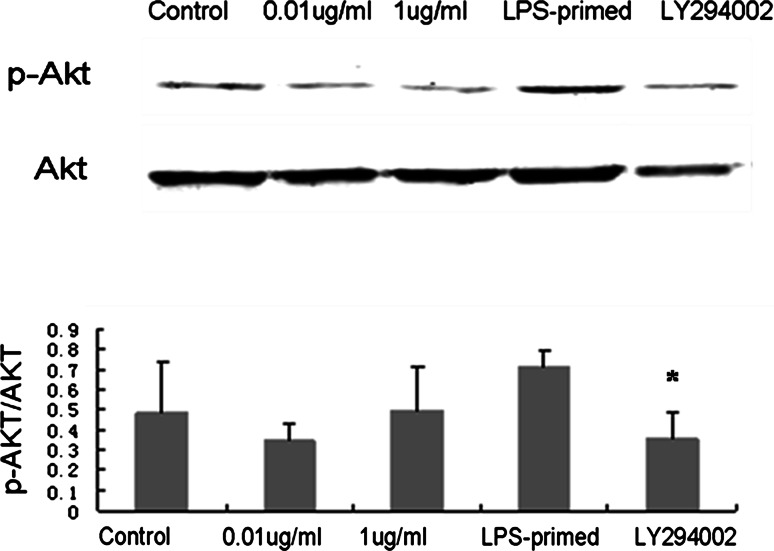
AKT phosphorylation in astrocytes with LPS preconditioning and LY294002 treatment. LPS preconditioning enhanced AKT phosphorylation in astrocytes. With the blockade of the PI3K/AKT pathway by LY294002, AKT phosphorylation was reduced in astrocytes with LPS preconditioning (compared with the LPS-primed group,* *P* < 0.05). (*Images* are representative of three independent experiments)

Immunofluorescence staining showed that LPS (0.01 and 1 μg/ml) tended to increase the cytoplasmic HMGB-1 expression in astrocytes (Fig. 5). After LPS preconditioning, HMGB-1 expression was enhanced with a diffuse staining in the cytoplasm (Fig. 5). As showed in Fig. 6, Western blot analysis demonstrated that LPS (0.01 and 1 μg/ml) was inclined to upregulate total HMGB-1 expression in astrocytes. To determine the protein level in the cytoplasm and nuclei, HMGB-1 in the cytoplasmic and nuclear extracts was detected. The results showed an increasing trend in the cytoplasm of LPS-treated (0.01, 1 μg/ml and LPS-primed) astrocytes, which implied a translocation of HMGB1 into the cytoplasm (Fig. 7a). As for the nuclear expression of HMGB-1, the results showed an inclined reduction in LPS-primed group, though no profound differences in statistics were exhibited (Fig. 7b). Using LY294002 for inhibition of PI3K pathway, the inhibitor could effectively suppress AKT phosphorylation in LPS-primed astrocytes (Fig. 8). Similarly, the expression of the cytoplasmic and nuclear HMGB-1 from LPS-primed astrocytes was reduced with the suppression of PI3K/AKT pathway (Figs. 5, 6, 7). These results suggested LPS-primed release of HMGB-1 into the cytoplasm was attenuated after blocking PI3K/AKT signaling using LY294002.

## Discussion

Low doses of LPS-induced protection against subsequent re-exposure to LPS in vivo and in macrophages in vitro (Kanaan et al. 2012; Zhang and Morrison 1993; Hirohashi and Morrison 1996). Moreover, during some brain injuries due to cerebral ischemia or autoimmune diseases, LPS preconditioning is protective as well (Bordet et al. 2000; Zimmermann et al. 2001; Buenafe and Bourdette 2007; Lin et al. 2009; Stevens et al. 2011; Vartanian and Stenzel-Poore 2011; Vartanian et al. 2011; Gesuete et al. 2012). Recent studies showed that protection conferred by LPS pretreatment involved the dampening of cellular immune responsiveness to brain injury, and was associated with reduced cellular infiltration and activation in the brain (Rosenzweig et al. 2004; Buenafe and Bourdette 2007; Vartanian and Stenzel-Poore 2011). Within CNS, the activation immune cells and/or inflammatory effector molecules from the neural cells, appeared to participate in the active modulation of the protective mechanism to LPS pre-exposure (Zhang and Morrison 1993; Shnyra et al. 1998; Vartanian and Stenzel-Poore 2011).

Reactive astrocytes play complex roles after stroke and brain injury. Glial scarring is deleterious, as this injury inhibits dendritic and axonal remodeling in neuronal circuits (Horner and Gage 2000; Rossi et al. 2007). However, reactive astrocytes might also secret beneficial mediators that encourage neuronal and vascular plasticity (Liberto et al. 2004). In the present study, we hypothesized that astrocytes might be involved in the cellular reprogramming of LPS preconditioning, contributing to the protective effects of these cells. Initially, we examined the responses of some cytokines and transcript factors in the present experimental paradigm, which were thought to be closely related to endotoxin tolerance. The data showed that TNF-α secretion and the activation of the transcript factor, NF-κb, in cultured astrocytes were down-regulated during LPS pretreatment. The suppressive changes of these inflammatory molecules are potent factors involved in the cellular attenuated responses to LPS re-exposure, which might be favorable for LPS-primed protective effects. Certainly, besides TNF-α, other cytokines such as IL-6 were also influenced by LPS pretreatment. Although previous studies demonstrated the partial down-regulation of IL-6 with repeated LPS challenge (Erroi et al., 1993), conversely, our data showed IL-6 levels from astrocytes were increased in response to LPS preconditioning. Although the down-regulation of TNF-α during LPS tolerance being well established in the present study, the responses of deferent mediators are eventually determined through various kinetic regulatory mechanisms and signal pathways, thereby governing multiple effects to harmful stimuli. In addition, whether LPS tolerance partially develops depends on the challenge model and cell types. Therefore, understandably, LPS tolerance did not show global down-regulation of signaling proteins and mediator production in astrocytes. The LPS-tolerant animals and cells might still upregulate specific genes and proteins in response to further LPS challenge (Vartanian and Stenzel-Poore 2011). In our study, we also dissected the responses of HMGB-1 from cultured astrocytes to LPS preconditioning. Based on the results, LPS preconditioning was inclined to enhance HMGB-1 expression in the cytoplasm, which suggested LPS priming involved in a release of HMGB-1 into the cytoplasm. Generally, HMGB-1 is regarded as an endogenous “alarmins,” which serves as danger signals and cytokines to promote the activation of the innate immunity in response to trauma, ischemia/reperfusion, or infection (Bianchi 2007). In stroke and brain injury, HMGB-1 might be released from ischemic neurons (Qiu et al. 2003), brain microglia (Kim et al. 2006), and activated monocytes/macrophages (Wang et al. 1999; Andersson et al. 2000), potentially mediating deleterious inflammatory responses (Andersson et al. 2000; Hayakawa et al. 2008, 2009). Recent studies have shown that reactive astrocytes release HMGB-1, which promotes neurovascular recovery after cerebral ischemia in mice (Hayakawa et al., 2009). Delayed HMGB-1 signaling in reactive astrocytes might promote neurovascular remodeling and neurite outgrowth (Huttunen et al. 2000; Treutiger et al. 2003). Thus, in this regard, HMGB-1 might play biphasic roles in the diverse pathophysiological processes. During the LPS-primed tolerance, most of studies have suggested the benefits of inducing tolerance in the context of inflammation milieu. The data obtained in the present study provided clues about HMGB-1 upregulation in this case. Although the precise function of HMGB-1 in LPS-priming astrocytes in vitro was not fully interpreted in current experiments, the results partially suggest that this molecule is actively involved in the reprogramming process, and might be a potential favor for LPS-priming protective effects in the CNS. Thus, to clarify the precise roles, and the interactions involving HMGB-1 from the astrocytic medium and neurons in vitro and in vivo are necessary for further studies (data not shown).

Addtionally, in the present study, we also probed to one potential mechanism, that is, the PI3K/AKT pathway, which controls HMGB-1 regulation during LPS tolerance. Previously, the activation of PI3K/AKT has been implicated to play a pivotal role in cytokine secretion and shown to negatively regulate the expression of NF-κb and inflammatory genes (Guha and Mackman 2002; Luyendyk et al. 2008). The PI3K activation suppressed LPS-induced lipoprotein lipase expression in J774 macrophages (Tengku-Muhammad et al. 1999) and nitric-oxide synthase in C6 glial cells (Pahan et al. 1999). Human and rat primary astrocytes were also negatively regulated by PI3K activation (Pahan et al. 2000). Nevertheless, in contrast to studies showing that the PI3K/AKT pathway negatively regulates the expression of inflammatory genes in macrophages, a recent study demonstrated that the PI3K/AKT pathway positively regulated NF-kb-dependent gene expression in HepG2 cells via phosphorylation and increased the transactivation activity of p65 (Luyendyk et al. 2008). It is proved that LPS tolerance has evolved as a complex counter-regulatory adaptive inflammatory response. Many signal pathways are recruited into the reprogramming process. Presently, the PI3K/AKT pathway has been implicated in the mediation of the LPS-primed reactive astrocytic upregulation and release of HMGB-1. We observed that using LY294002 to block the PI3K pathway inhibited LPS-primed HMGB-1 release from astrocytes, which was similar to the changes of TNF-α, IL-6, and NF-kb. Previous studies have suggested that the PI3K pathway remains open in LPS tolerance. Our results revealed the positive regulation of HMGB-1 expression and pro-inflammatory mediators in LPS-pretreated primary astrocytes. This pathway might represent an important flexible mechanism to regulate the activity of HMGB-1, and potentially provides a candidate for modifying and augmenting the protective effects in astrocytes in response to LPS preconditioning after brain inflammation or injuries. Nonetheless, considering further perplexities of LPS tolerance in vivo, and the internal milieu is distinct from that in vitro, determining how HMGB-1 is co-regulated in other cell types or signal pathways in the cerebral brain requires more profoundly unraveling.

In conclusion, with LPS preconditioning, cortical astrocytes evolved an adjusted cellular response in vitro, and the PI3K/AKT pathway positively regulated LPS-primed release of HMGB-1 from astrocytes. These findings provide insights into the mechanisms underlying LPS priming and the beneficial actions of resident cells in response to diverse brain insults.
